# Primary school children's health and its association with physical fitness development and health-related factors

**DOI:** 10.3934/publichealth.2024001

**Published:** 2023-12-04

**Authors:** Gerhard Ruedl, Armando Cocca, Katharina C. Wirnitzer, Derrick Tanous, Clemens Drenowatz, Martin Niedermeier

**Affiliations:** 1 Department of Sport Science, University of Innsbruck, Furstenweg 185, 6020 Innsbruck, Austria; 2 Department of Human Movement Studies, University of Ostrava, Dvořákova 138/7, 70200 Ostrava, the Czech Republic; 3 Department of Research and Development in Teacher Education, University College of Teacher Education Tyrol, Pastorstraße 7, 6010 Innsbruck, Austria; 4 Research Center Medical Humanities, Leopold-Franzens University of Innsbruck, Innrain 52, 6020 Innsbruck, Austria; 5 Division of Sport, Physical Activity and Health, University of Education Upper Austria, Kaplanhofstraße 40, 4020 Linz, Austria

**Keywords:** well-being, conditional skills, physical activity, youth, media consumption, socio-economic factors

## Abstract

The health status (HS) of children is influenced by a variety of factors, including physical fitness (PF) or social and environmental characteristics. We present a 4-year longitudinal study carried out with 263 primary school children. PF was assessed yearly using the German Motor Performance Test 6–18. Demographic data, leisure time behavior and socioeconomic factors were collected using questionnaires for children and parents. Based on parents' ratings in year 4, children were categorized as either “very good health status” (VGHS) or “good health status or below” (GHSB). Children with VGHS (73%) showed a larger improvement of global PF (*p* < 0.001), a significantly higher proportion of being/playing outside (*p* < 0.001), significantly lower proportions of overweight (*p* < 0.001), of media availability in the bedroom (*p* = 0.011) and of daily media consumption > 2 h (*p* = 0.033) compared to children with GHSB. Regarding socio-economic factors, children with VGHS revealed significantly fewer parents with lower education (*p* = 0.002), lower physical activity levels (*p* = 0.030) and lower migration background (*p* < 0.001). Physical fitness (*p* = 0.019) and outdoors exercising (*p* = 0.050) were the only variables to provide significantly higher chances of perceiving one's own health as very good when tested within a complex model including all the variables studied in this work. Considering the little focus on PF in the current Austrian physical education curriculum and the favorable environmental features of the Tyrolean region, more emphasis should be given to promoting didactical and pedagogical approaches that allow schoolers to be active in the nature.

## 1. Introduction

A positive health condition is essential at all ages, even more at early stages of life, since healthy children have higher opportunities to develop properly and fully, thus achieving their full potential (World Health Organization, 2023) [1]. Although there are several scientific approaches to assessing people's and children's health status, individual self-perception has grown to a widely used method due to several advantages. Self-perception tools are easy to apply, encompass wider strata of the population at low costs and have been shown to be a valid assessment strategy [2]. Self-rated health (SRH) is commonly measured by the single-item question, “Would you say your health is...”, rating their status of general health with a 4- or 5-point Likert response scale, indicating a graded level of health status [3],[4]. This approach includes multiple health-related aspects, e.g., physical, psychological and social health, which are not available to external observers [4]. SRH in children is influenced by a variety of factors: Sociodemographic, body mass index (BMI) and body weight perception, self-esteem, diet, substance abuse, social factors (socio-economic status, child-parent relationships, school achievement), physical activity (PA) [5],[6], physical fitness (PF) and sedentary behavior [7],[8]. For instance, a study on German children and adolescents highlighted that social (parents' socioeconomic status and migration background), behavioral (PA habits, including leisure-time and outdoors exercise) and physical parameters (weight status) may interact with SRH [9]. Media consumption was also found to be significantly associated with children's perceived health, thus requiring it to be considered in the complex structure of SRH-related variables [10]. Regarding PF, this variable is known to have a strong impact on SRH starting from early ages [11]. A higher PF positively impacts cardiometabolic disease risk, adiposity, mental health, cognition and bone health in children [12],[13], while a lower PF in youth is significantly associated with all-cause and cancer-associated mortality later in life [14],[15]. The association of SRH with sedentary behaviors and PA, both variables strongly linked to PF, was recently investigated by Zhang T et al. [4] through a systematic review. The authors reported strong evidence for a negative relationship between SRH and sedentary behavior, in addition to a positive relationship with PA among children and adolescents. Beyond the studies that have investigated the association of SRH with a series of individual and socio-behavioral variables, some authors have delved into how different levels of perceived health may correlate with other health-related outputs. For instance, Krause L and Mauz E [16] proposed to dichotomize children's health levels into very good/good and moderate or lower, thus comparing how these two groups may differ in terms of physical, mental or social health. However, other studies pointed out that differences may be found even among children with good SRH and above. For instance, a study by Elissa K et al. [17] found that the majority of their participants perceived their health as at least good; still, those with higher reported health status showed significantly better results in the other health-related variable assessed, i.e., sense of coherence. In line with these findings, a full sample of Finnish children aged 7 to 14 years old reported high SRH [18]. Nonetheless, logistic regression analyses showed that sedentary behaviors were significantly more common in the portion of the sample reporting less high health status, whilst children with the highest SRH were more likely to be more active [18]. In general, the vast majority of these studies have focused on a cross-sectional design, allowing us to understand the relation between SRH and other variables in a particular time point. On the other hand, only a small stream of research has tried to investigate trends over a longer period. Among them, Padilla-Moledo C et al. [19] investigated cross-sectional and longitudinal associations between PF components and SRH among a cohort of Spanish youth aged 8-18 years. These authors found that cardiorespiratory fitness and global fitness (i.e., the combination of all the individual fitness components, considered altogether) were positively associated with SRH in children at baseline and two years later [19]. Recently, Hanssen-Doose A et al. [20] demonstrated a positive predictive value of coordination and muscular fitness on SRH at a later stage among German youth aged 10 years and older. However, these works have either covered a relatively small time period (up to two years, which may not be sufficient to fully encompass the physical and social-cognitive changes that happen during the pre-adolescence phase), or have focused only the relation between SRH and a specific variable (such as, PF, hence not considering other elements that could potentially impact children's perception of their own health). Therefore, we aimed to compare changes in PF and socioeconomic factors over the whole primary education period (four years) based on different levels of SRH and to examine which changes in the above factors may be associated with higher perceived health in Austrian children from Tyrol.

## 2. Materials and Methods

### 2.1. Participants

**Figure 1.**
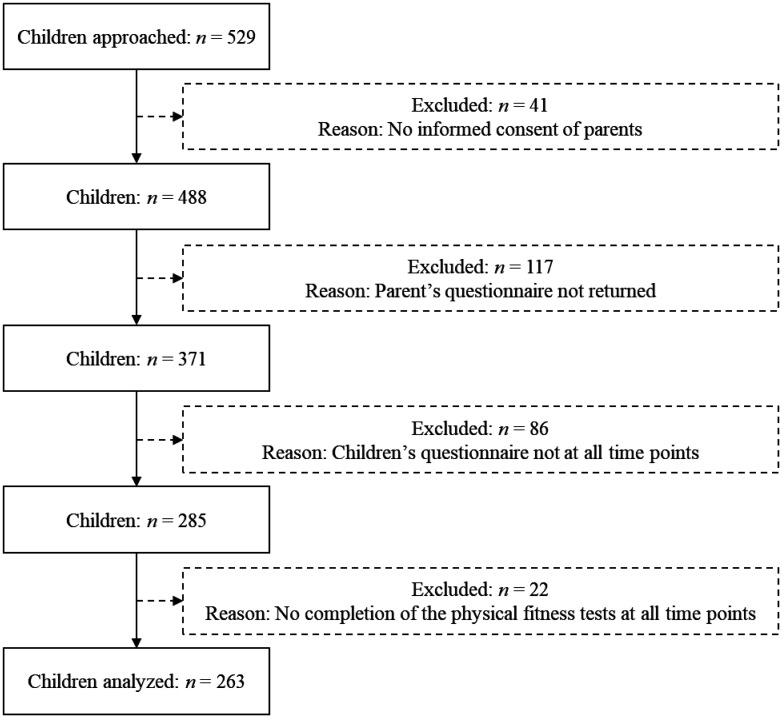
Flowchart of the study participants.

There are currently 361 primary schools in the federal state of Tyrol (Austria). From this pool, twenty randomly selected schools were sent information about the research and their principals were asked for permission to access their facilities. The final sample consisted of a total of 529 students from 15 primary schools (those whose principals agree to participate) located in Tyrol, Austria. The samples included four schools with 163 children located in an urban setting. The participants underwent testing sessions twice per year (every spring and every autumn) during the four years of primary education. All measured variables were tested at each time point every year. Inclusion criteria for participation in the study are described as follows: a) Completion of PF tests at all time points; b) return of the children's questionnaire at all time points; and c) parental return of the specific questionnaire made for them. At the end of the observation period, the samples were composed of 263 participants with 44.9% (118) girls and 55.1% (145) boys and mean age of 6.9 (0.5) years in the first year (Figure 1).

The study was conducted according to the ethical standards of the Declaration of Helsinki (as amended in 2013) and was approved by the educational board for Tyrol and the Institutional Review Board for Ethical Issues of the institution. All parents received written information about the study and provided written informed consent for themselves and their children before taking part in the study.

### 2.2. Instruments

#### 2.2.1. Children's anthropometric measurements

The measurement of height and weight, along with the PF testing, was carried out by university students previously trained by the research team. As instructed during the training period, they followed the guidelines included in the testing manual available for the PF tests, which included weight and height measuring procedures. At each measurement session, one of the members of the research team provided support and supervision. Measures were taken by means of a mobile stadiometer “Seca 217” (Seca, Hamburg, Germany) with an accuracy of 0.1 cm and a calibrated scale “Grundig PS 2010” (Grundig AG, Neu-Isenburg, Germany) with an accuracy of 0.1 kg, respectively. Children were asked to wear sports clothes and take off their shoes. BMI was then calculated using the obtained data. Using the categorization proposed by Kromeyer-Hauschild K et al. [21], five BMI groups were created: “anorexic”, “underweight”, “normal weight”, “overweight” and “obese” (BMI > 90^th^ percentile). Further classification of the participants consisted in distributing them into either an overweight group (BMI > 90^th^ percentile) or a non-overweight one (BMI ≤ 90^th^ percentile). At the end of the observation period, a single variable with two levels was calculated based on the combination of the weight status at the four time points: Level 0, i.e., children who were never classified as overweight at any time point; and level 1, i.e., children who fell in the overweight category at least once.

#### 2.2.2. Physical fitness

The German Motor Performance Test (GMT) 6–18 was carried out for measuring PF at each time point [22]. As explained above, trained students carried out all the testing with the support of a research supervisor and the testing manual available together with the GMT. The GMT 6–18 is a standardized test battery composed by 8 subtests, each of them measuring a different component of the PF [22]. More specifically, speed is evaluated with a 20-m sprint test; coordination and precision by means of balancing backwards on three 3 m-long beams with different widths (3, 4.5 and 6 cm); coordination under time pressure, by means of jumping sideways over a middle line for 15 s; flexibility with the stand-and-reach test (participant standing on a long bench and bending the upper body forward as far down as possible with extended legs; the difference between the fingertips and the top of the bench is taken as the measure); muscular endurance, by means of the 40-s push-up test and the 40-s sit-up test; muscular power with the standing long jump test; and cardiorespiratory fitness by means of the 6-minute run test. Following the guidelines for the GMT battery of tests, specific instructions were given to participants at each testing station, and the children were allowed to carry out practice trials prior to formally measuring their performance [22]. The GMT has been previously validated and showed satisfactory inter-rater reliability (*r* = 0.95) and test-retest reliability (*r* = 0.82) [22]. Trained students from the Bachelor of Science in Physical Education led all testing sessions, which were carried out indoors following the protocol proposed by Bös K et al. [22].

Furthermore, a global PF score was calculated by means of z-transformation of the mean values and standard deviations of the subtests obtained in the first year of measurement. In a similar manner, the global PF score was also calculated using the subtest scores and standard deviations obtained in the fourth (last) year. In order to ensure uniformity of scores for the calculation of the z-transformed variables, the values from the 20-meter sprint test were multiplied by -1; this was necessary due to the fact that speed was the only test in which lower raw values meant better performances. Following the suggestion by Utesch T et al. [23], who found contrasting results for the balancing and the flexibility tests, these two subtests were excluded from the final calculation of the global PF score. The global PF score was then obtained by computing an overall mean value of the six z-transformed subtests. Changes in global PF from year one to year four were assessed by calculating the difference between the global PF scores at the last time point and the first time point. Results above zero meant a development of the overall PF over the four years, whereas PF decreased when that difference was below zero.

#### 2.2.3. Children's questionnaire

Children were asked to fill in a standardized questionnaire based on a previous study on a yearly basis containing 17 items [24]. This questionnaire assessed different sociodemographic variables (sex, migration background), behavioral aspects (engagement in PA both at a sports club level and freely as a part of the leisure time) and social/environmental features (availability of electronics in the bedroom, smartphone ownership, use of communication apps, such as Whatsapp, from/to-school transportation means). Response options were predominantly dichotomous (e.g., “yes” or “no” for availability of electronics in the bedroom, smartphone ownership, Whatsapp). No total score was calculated for the questionnaire. Instead, once data was collected for each of the four years of observation, categories per each variable separately were computed by means of combining the information from the four measuring sessions. Binary variables were created as follows: For the information on electronics in the bedroom, smartphone ownership, Whatsapp usage and engagement in PA at sports clubs, a value of 0 means never during the four years, whilst a value of 1 means at least at one point in the four years. For the transportation variable, a value of 0 was given if children responded that they never used active means during the four years, whilst those children who were actively moving from and to school at least one out of the four years were assigned the value of 1.

#### 2.2.4. Parents' questionnaire

A questionnaire was administered to the parents of the children who participated in the study to collect information on their educational background and PA habits. Data was gathered separately for fathers and mothers. In terms of education, they were asked to disclose their highest completed education, the options being middle school, high school with business specialization, high school with engineering specialization, general high school diploma, bachelor studies and other. Additionally, parents had to specify the frequency of their PA during a typical week, the options being: No PA, irregular participation in PA, regularly 1–2 times per week or regularly 3 or more times per week. Parents' education was successively coded: A value of 1 was given in case at least one parent completed upper secondary education, such as high school, or higher; a value of 0 was assigned when none of the parents had obtained any upper secondary education diploma (or higher) [25]. Regarding PA, following the protocol from a previous study [26], active habits were coded with a value of 1 in the cases in which at least one parent carried out regular PA in a typical week (i.e., at least regularly 1–2 times per week); or with a value of 0 if both had responded to either not be active at all or only irregularly. In the case of single parents or if information could only be collected from one parent, their responses were coded as above explained without combining them with the second parent's data. Additionally, parents filled in information on their children's screen-time habits, specifically the daily amount of time spent on TV or other screen-based electronics, the possible responses being: Less than 1 hour; 1 or 2 hours; 3 or 4 hours; or more than 4 hours. Finally, parents were asked to provide information on their children's outdoors playing time per week. The answer was given on a list of options as follows: “my child plays mainly inside at home”; “my child plays outdoors 1 or 2 days per week outdoors”; “3 or 4 days per week”; or “5 and more days per week”. For the purpose of this study, these data were converted into binary variables as follows: for the screen time frequency, a value of 0 was assigned when children spent on a screen 2 hours/day or less; a value of 1, if 2 hours/day or more. In the same way, playing outdoors was coded as 0 if the child played less than 5 days/week; or it was coded as 1 if they played outdoors 5 days/week or more in accordance with a previous study [27]. Regarding perceived health status, it is recommended that parents provide data on their children rather than the children themselves [16],[28] as children younger than 11 years old tend to be less reliable when reflecting on their own health [20]. Parents rated their child's health with the question: “Would you say the health of your child in general is ...”. A 5-point Likert scale (very good, good, moderate, poor, very poor) was used, in line with previous studies [16],[20]. Responses were further dichotomized in two categories: “very good health” (VGHS) and “good health and below,” (GHSB) due to a low number of children with moderate, poor and very poor perceived health in this study sample.

### 2.3. Data analysis

All analyses were conducted using SPSS Statistics version 26 (IBM, New York, United States). The overarching research question was which factors are associated with the self-reported health status of the children. The role of the development of PF as a potential factor was of special interest. The identification of the factors associated was done by a two-step analysis: The first step was the identification of associated factors with health status using a simple analysis of group differences. The second step consisted of a multiple analysis of the associated factors identified as significant in the first step.

In the first step of the analysis, differences between children with very good health status and children with good health status or below were analyzed for each variable separately. Depending on the measurement scale of the variable and the normality distribution (according to Shapiro Wilk test), *t*-tests for independent samples (continuous, normally distributed variables), Mann-Whitney-U-tests (continuous, non-normally distributed variables), or Pearson's *χ²* tests (frequencies of dichotomous variables) were used. In the analysis of the group differences, health status (children with very good health status vs. children with good health status or below) was the independent variable and the analyzed factors (e.g., development of PF, age, or sex) were the dependent variables. Cohen's d was calculated as an effect size in the analysis of group differences between children with very good health status and children with good health status or below for all continuous variables. The conventions small (0.2), medium (0.5) and large (0.8) were used [29]. Odds ratio (OR) was calculated in the analysis of group differences between children with very good health status and children with good health status or below for all dichotomous variables (e.g., sex, migration background or weight status).

In the second step of the analysis, all variables that were found significant (*p* < 0.05) in the first step were used as independent variables in a multiple logistic regression analysis. The dependent variable was “health status group” with the two levels: “children with very good health status” and “children with good health status or below”. OR was given as an effect size for all independent variables [30]. *P*-values of less than .05 were considered significant (two-tailed). Unless otherwise stated, values represent mean (SD) and relative (absolute) frequencies.

## 3. Results

Out of all children, 73.4% (193) were in the group of children with very good health, and 26.6% (70) in the group of children with good health or below. The distribution of the original health status categories was 73.4% (193) in very good health, 23.2% (61) in good health, 3.4% (9) in moderate health and 0% (0) in both poor health and very poor health. The sample's sex distribution was 44.9% (118) girls and 55.1% (145) boys. The mean age was 6.9 (0.5) years in the first year and 9.9 (0.6) years in the last year.

### 3.1. Group comparison

The first step of the analysis contained the analyses of differences between children with very good health status and children with good health status or below for each variable separately. Significant group differences between the group with very good health and the group with good health or below in physical fitness were found in age and in global PF (including some subtests of PF) (Table 1). The group with very good health status showed a significantly younger mean age and a larger mean improvement of global PF (significant subtests: 20 m-sprint, push-ups, sit-ups, standing long jump and 6-minute run) compared to the group with good health or below.

**Table 1.** Group differences in mean age and mean development of physical fitness between the group of children with very good health status and children with good health status or below.

Variable	Very good health status (*n* = 193)		Good health status or below (*n* = 70)		*p*	*d*
	Mean	(*SD*)	Mean	(*SD*)		
Age (year 1) [years]	6.8	(0.6)	7.1	(0.5)	**0.002** ^b^	**-0.44**
Change in physical fitness subtests (year 4–year 1)				
Δ z-score total (6 subtests)	1.5	(0.6)	1.1	(0.7)	**<0.001** ^a^	**0.65**
Δ 20-meter sprint [s]	-0.6	(0.4)	-0.5	(0.4)	**0.001** ^b^	**-0.37**
Δ Balancing backward [n steps]	10	9	9	10	0.297^a^	0.15
Δ Jumping sideways [n jumps]	15	7	14	6	0.360^b^	0.08
Δ Stand-and-reach [cm]	-0.3	5.9	-0.4	6.1	0.952^a^	0.01
Δ Push-ups [n]	5	5	3	6	**0.004** ^a^	**0.41**
Δ Sit-ups [n]	9	5	6	6	**0.006** ^a^	**0.43**
Standing long jump [cm]	27	15	18	17	**<0.001** ^a^	**0.56**
Δ 6-minute run [m]	134	139	59	139	**<0.001** ^a^	**0.55**

Note: ^a^: *t*-test for independent samples, ^b^: Mann-Whitney-U-test, d: Cohen's d, Δ z-score total: difference in the average z-value between the last time point in the fourth year and the first time point in the first year of primary school. Higher Δ z-score total values indicate larger developments. Bold values indicate comparisons with *p* < 0.05.

**Table 2.** Group differences in sex, weight status, physical activity and media consumption between the group of children with very good health status and children with good health status or below.

Variable	Very good health status (*n* = 193)		Good health status or below (*n* = 70)		*p*	*OR*
	%	(*n*)	%	(*n*)		
Sex					0.655	
female	44.0%	(85)	47.1%	(33)		1 (ref)
male	56.0%	(108)	52.9%	(37)		1.13
Weight status					**<0.001**	
never overweight	81.3%	(157)	51.4%	(36)		1 (ref)
at least once overweight	18.7%	(36)	48.6%	(34)		**0.24**
Sports club participation					0.144	
never	9.3%	(18)	15.7%	(11)		1 (ref)
at least once	90.7%	(175)	84.3%	(59)		1.81
Being/playing outside					**<0.001**	
at least once less than 5 days/week	48.5%	(80)	76.6%	(49)		1 (ref)
always 5 days/week or more	51.5%	(85)	23.4%	(15)		**3.47**
Way to school					0.244	
never by feet, bike, scooter	22.3%	(43)	15.7%	(11)		1 (ref)
at least once by feet, bike, scooter	77.7%	(150)	84.3%	(59)		0.65
Media consumption						
TV/PC in pupils' room					**0.011**	
never	24.4%	(47)	10.0%	(7)		1 (ref)
at least once	75.6%	(146)	90.0%	(63)		**0.35**
Hours TV/PC per day					**0.001**	
always 2 hours/day and below	89.1%	(172)	72.9%	(51)		1 (ref)
at least once > 2 hours/day	10.9%	(21)	27.1%	(19)		**0.33**
Own smartphone					0.119	
never	19.7%	(38)	11.4%	(8)		1 (ref)
at least once	80.3%	(155)	88.6%	(62)		0.53
WhatsApp					0.102	
never	26.9%	(52)	17.1%	(12)		1 (ref)
at least once	73.1%	(141)	82.9%	(58)		0.56

Note: *OR*: odds ratio, ref: reference category. Bold values indicate comparisons with *p* < 0.05. Not all frequencies sum up to *n* = 263 due to missing values.

The analysis of group differences between the group with very good health and the group with good health or below in sex, weight status, physical activity and media consumption is shown in Table 2. Significant differences between the group with very good health and the group with good health or below were found in weight status, being/playing outside and in two media consumption variables. Children in the group with very good health status had a significantly higher chance to be never overweight, to always be/play outside 5 days/week or more, to never have a TV/PC in their bedroom and to always use the TV/PC 2 hours/day or below compared to the group with good health or below.

Significant differences between the group with very good health and the group with good health or below were found in parental education, parental physical activity and migration background (Table 3). Children with a very good perceived health status showed significantly lower proportions of both parents with lower secondary education or below and of both parents not/irregular physically active compared to the group with good health or below. Furthermore, children with a very good perceived health status showed a significantly lower proportion of migrants compared to the group with good health or below.

**Table 3.** Group differences in socioeconomic factors between the group of children with very good health status and children with good health status or below.

Variable	Very good health status (*n* = 193)		Good health status or below (*n* = 70)		*p*	*OR*
	%	(*n*)	%	(*n*)		
Parental education					**0.002**	
both parents lower secondary education or below	32.3%	(61)	53.6%	(37)		1 (ref)
at least one parent upper secondary education or above	67.7%	(128)	46.4%	(32)		**2.43**
Parental physical activity					**0.030**	
both parents not/irregular physically active	24.9%	(48)	38.6%	(27)		1 (ref)
at least one parent regularly physically active	75.1%	(145)	61.4%	(43)		**1.90**
Migration background					**0.001**	
non-migrants	80.8%	(156)	60.0%	(42)		1 (ref)
migrants	19.2%	(37)	40.0%	(28)		**0.36**

Note: *OR*: odds ratio, ref: reference category. Bold values indicate comparisons with *p* < 0.05. Not all frequencies sum up to *n* = 263 due to missing values.

### 3.2. Multiple logistic regression analysis

The results of the multiple logistic regression analysis are displayed in Table 4. The dichotomous variable health status was the dependent variable. All variables that showed significant differences in the simple analyses were included as independent variables. The analysis indicated the development of global physical fitness according to the Δ z-score total and being/playing outside as significant factors being associated with the dependent variable health status. A larger increase in global physical fitness over the four years was associated with higher odds to be in the group with very good health compared to the group with good health or below (*OR* = 1.97). Similarly, being/playing outside always five days/week or more was associated with a higher chance to be in the group with very good health (*OR* = 2.10). The remaining factors including all media consumption variables showed the same direction of association as in the simple analysis (tests on group differences reported in sub-section 3.1) but were not significant in the multiple analysis.

**Table 4.** Results of the multiple logistic regression analysis with health status as the dependent variable (*n* = 224).

Variable	*B*	Standard error of B	*OR*	*OR* 95% *CI* lb	*OR* 95% *CI* ub	*p*
Age (year 1) [years]	-0.61	(0.36)	0.54	0.27	1.09	0.088
Δ z-score total (6 subtests)	0.68	(0.29)	**1.97**	1.12	3.45	**0.019**
Migration background	-0.03	(0.40)	0.97	0.44	2.15	0.943
Parental education	0.37	(0.35)	1.45	0.72	2.91	0.293
Parental physical activity	0.30	(0.36)	1.34	0.66	2.73	0.414
Weight status	-0.73	(0.37)	0.48	0.23	1.00	0.052
Being/playing outside	0.74	(0.38)	**2.10**	1.00	4.41	**0.050**
TV/PC in pupils' room	-0.62	(0.51)	0.54	0.20	1.48	0.231
Hours TV/PC per day	-0.77	(0.43)	0.46	0.20	1.08	0.076
Constant	4.60	(2.83)	99.92			0.104

Note: *B*: unstandardized regression coefficient, *OR*: odds ratio, *OR* 95% *CI*: 95% confidence interval of the odds ratio, lb: lower bound, ub: upper bound. Δ z-score total: difference in the average z-value between the last time point in the fourth year and the first time point in the first year of primary school. Bold values represent significant factors, Nagelkerkes *R²*: 28.1%

## 4. Discussion

We aimed to compare changes in different health-related variables over the four years of primary education based on self-reported health status level and to analyze associated factors with the health status of primary school children with a special focus on the development of PF over the four years of primary school.

### 4.1. Comparison of changes in health-related variables

In accordance with our results, Padilla-Moledo C et al. [19] analyzed some children and found a positive association between global fitness and cardiorespiratory fitness and SRH at baseline and after two years. Generally, lower cardiorespiratory and muscular fitness at young ages is associated with adiposity, lower cardiometabolic health and lower bone health later in life [11]–[13]. The authors highlight that enhancing PF during the primary education period may be extremely important when aiming at increasing individual health. Increases in cardiorespiratory and muscular fitness of primary school children can be achieved using several pedagogical models and can contribute to increasing overall PA engagement [31]–[33]. Indeed, PA is important for children's health and wellbeing. There is evidence that higher levels of PA are positively associated with SRH of all age groups [4],[34]–[36]. In a two-year longitudinal study, increasing SRH predicted both PA and maintaining sports activity among Norwegian adolescents [30]. However, there seems to be evidence that this association is bidirectional [37]. A particular PA condition that contributes to health is that carried out during leisure time [9]. In our study, children with VGHS reported more than a two-fold higher proportion of being/playing outside for five or more days per week compared to children with GHSB (52% *vs*. 23%), which is in line with previous findings. Regarding body weight, there is wide evidence that a lower SRH is associated with higher body weight and negative body weight perception [8],[38]–[39]. Similar to the existing literature, never being overweight during the four years of primary school was significantly associated with VGHS. Meland E et al. [40] found that increased body mass had unfavorable effects on SRH in a two-year longitudinal study with 1225 Norwegian high school students. In addition, positive SRH was associated with a leaner body after two years, as well as with a beneficial BMI change during the two years of the observation period [40]. A study on adolescents aged 12 to 17 years old pointed out that those perceiving their weight status as “just right” also tended to perceive their own health as “very good” or “excellent” [39]. Moreover, these adolescents seemed to be more prone to assuming fruits and vegetables compared to their peer who reported to be “underweight” or “overweight” [39]. Our study adds to this knowledge; however, it also suggests that differences may be significant even in children with good perceived health compared to those with excellent SRH. With regard to electronic devices and their usage, children with VGHS showed lower proportions of media availability in the bedroom and daily media consumption >2 h. In line with this, in a study carried out in Canada, adolescents who exceeded the recommendation of a maximum of 2 h/day of screen time were found to have 30% greater odds of reporting suboptimal SRH, and 30%–50% greater odds of reporting suboptimal mental health [36]. According to Matin N et al. [41], low screen time along with high PA leads to adolescents having higher chances to perceive themselves as in good health (OR 1.37) and to feel satisfied with their life (OR 1.43). On the other hand, exceeding screen time recommendations led to 60% higher chance of having poor fitness compared to youth maintaining screen time under 2 hours per day [42]. Finally, our analysis showed that VGHS was significantly less frequent in kids whose parents reported lower education and lower PA level. In addition, the proportion of migration background was lower in children with VGHS. Higher parental education might be associated with more sports participation and higher involvement in recreational activities and likely with a higher awareness of the health effects of PA [43]. The importance of parental educational status was similarly shown for healthy nutritional behavior in children with higher educated parents [43]. Okamoto S [44] found a positive association between adolescents' SRH and subjective economic status among a cohort of more than 3000 Japanese adolescents. In addition, adolescents with parents in middle and high-income groups were more likely to report better SRH than those with parents in the low-income category [44].

### 4.2. Factors associated with self-perceived health status

Although many of the variables considered in this study have shown significant differences when comparing children perceiving their health as very good compared to their peers with lower perceived health, the assessment of an explanatory model that included them all at once led to different findings. In fact, as our results show, only PF and outdoors PA significantly increased the chances of children in our sample to perceive their health as very good. As previously discussed, many studies have approached the association between perceived health and socio-economic, environmental, or behavioral variables, most of them agreeing on the fact that these variables significantly contribute to SRH. Nonetheless, none has tried to track all these variables over a period of time and to assess how their changes may interact and influence SRH within a complex model. The clear association between a good PF and feeling healthy is well known in research; hence, it is no surprise that this variable plays an essential role in increasing one's perception of their wellbeing. Furthermore, outdoors leisure-time PA has been reported as highly beneficial in many studies. For instance, although carried with older participants, a study by Kerr J et al. [45] reported that longer outdoors PA time was associated with significantly higher self-reported functioning. This was further confirmed by a systematic review, highlighting that nature-based PA is effective to improve mental and physical health even in individuals with mental issues [46]. Sando OJ [47] used a multilevel approach to study the relation between PA, outdoor environment and children's health, pointing out that playing in an active way in natural settings is associated with better health outcomes. An interesting point is provided by Pasanen TP et al. [48], who carried out a study on a large sample including individuals from youth to elderly. The authors emphasize that being in a natural outdoor environment seems to bring additional value to the existing benefits of PA. An essential aspect of their findings is that they controlled for confounding variables, such as socio-economic status and other related factors. This may help to understand and justify the outcomes presented in our study, since, in line with those authors, we found a significant impact of outdoors exercise whereas socio-economic factors were excluded from the final model. Perhaps, although all of these factors contribute individually to one's perception of their own health, an advantage of PA is that it can be performed in any moment and regardless of a person's financial condition, family status, etc. This is more evident regarding outdoors PA: In fact, participation in sports clubs or using other facilities may require the financial ability to purchase a membership/entry ticket. On the contrary, anyone can freely exercise in the nature with no need of financial sources.

### 4.3. Limitations

The most critical limitation of the present study is the use of self-reported data, which may lead to potential risks, such as untruthful responses, difficulties in properly or precisely recalling events or social desirability biases. Furthermore, the categorization of all variables, mostly in dichotomous ones, may oversimplify aspects that, in reality, are more complex and would require a deeper analysis, especially in regard to parents' PA habits: The frequency of their weekly PA may provide a general overview of their lifestyle, yet, variables such as intensity and duration of each PA session are also important and may have an impact as well. The major strengths of this study are its longitudinal design along with the final sample size, both being not common especially in primary education children. Also, this study brings information in an area that currently has gaps, i.e., children's health associated with several physical, social and environmental variables and their change over several years. Although with the limitations presented above, the wide range of variables included allows to have a greater overview of the intertwined impact of different individual areas on children's perception of their health condition.

## 5. Conclusions

The current Austrian curriculum for primary school makes little reference to specific contents that, instead, are cornerstones for improving students' health in the short and long term. Indeed, physical fitness and exercise, although included in the “Movement and Sports” domain of schools plans, present several shortcomings, among which the lack of specialists for the teaching of physical education (PE), and a curriculum mostly focused on motor-skill development and psychological and social development through exercise, do not endorse the role of fitness or any didactical progression/pedagogical strategy to improve it over the primary school period, despite their well-known efficacy at this stage [31],[32]. Indeed, children's natural need to move may conceal this inadequacy in early stages of education; in addition, the role of motor-skill development, which is an integral part of the current Austrian PE curriculum, towards the structuring of active habits and increasing PF in children has been underlined in previous works [49], and may also contribute to “hiding” the lack of PF-focused units in primary education PE. Nonetheless, data from Austrian adolescents – showing only 17% are sufficiently active [50]–suggest that schools may need to start building health literacy from early grades, as well as to add more didactical blocks dedicated to PF starting from early educational levels. In addition, considering the favorable environmental characteristics of the Tyrolean region, increasing outdoors activities during PE may provide with added benefits not only for kids' physical health, but also their mental wellbeing and adherence to exercise. Finally, independent media usage in children has increased over the last decade and is now very common among primary school students [51]. Although, the current Austrian curriculum for primary schools proposes only to integrate this matter into the subjects, more emphasis to media and digitalization will be given in the new curriculum in force from the academic year 2022/2023. Our study may provide an insight on the longitudinal association between general health and active/inactive behaviors, therefore providing ideas for the direction that primary school curricula may take in the future in order to promote youth's wellbeing.

## Use of AI tools declaration

The authors declare they have not used artificial intelligence (AI) tools in the creation of this article.
